# *Eucalyptus* transcriptome analysis revealed molecular chaperones highly expressed in xylem

**DOI:** 10.1186/1753-6561-5-S7-P109

**Published:** 2011-09-13

**Authors:** Danieli Gonçalves, Jorge Lepikson-Neto, Marcela Salazar, Leandro Nascimento, Eduardo Camargo, Wesley Marques, Gonçalo Pereira, Carlos Ramos

**Affiliations:** 1 Laboratório de Bioquímica de Proteínas, Dep. de Química Orgânica - Instituto de Química, Universidade Estadual de Campinas, UNICAMP, Brazil; 2 Laboratório de Genômica e Expressão, Dep. de Genética, Evolução e Bioagentes - Instituto de Biologia, Universidade Estadual de Campinas, UNICAMP, Brazil

## Background

Plant development is very plastic, being coupled to environmental cues. As sessile organisms, plants must be able to respond rapidly to environmental stresses such as changes in temperature and salinity, heavy metals and water deficit. Efficient stress response systems are prerequisites for plant survival and productivity [1].

Molecular chaperones (or Heat Shock Proteins – HSP) compose a ubiquitous class of proteins involved in cellular protein quality control (PQC) and homeostasis. They play a critical role in folding and degradation of polypeptides, and therefore, in maintenance and modulation of cellular pathways, which are dependent of function (correct folding) and availability (stability and degradation) of involved proteins, under normal and stress conditions [2].

Genetics and proteomics studies of wood formation have highlighted some chaperones up-regulated in xylem of *Eucalyptus*, *Pinus* and *Populus* species, stating that they may play an important role in cell wall formation and xylem development [3,4].

Different species of *Eucalyptus* are known for their superior performance in growth, wood quality and resistance to different types of stress [5]. Such characteristics are probably driven by distinct gene expression coordination in xylogenesis. *Eucalyptus grandis* is one of the most planted species in the world due to its rapid growth, wide adaptability and wood quality. *Eucalyptus globulus* wood has higher S/G ratio which provides high yields in cellulose extraction [6].

Lignin extraction consumes large quantities of chemicals and energy, and many efforts have been made to improve this process by modifying lignin content or composition in trees, in order to reduce lignin content or make it easier to extract. Results have been achieved by supplementation and genetic modification [7,8].

This study aims to identify chaperones possibly involved in wood formation and quality of wood for pulp and paper industries.

## Methods

The RNA-Seq reads were produced from two xylem libraries for comparison between species (*Eucalyptus globulus* and*E. grandis*), and from two libraries for evaluating flavonoids supplementation (*E. urograndis* supplemented with naringenin and narigenin-chalcone).

Reads were aligned against the assembled unigenes using SOAP2 aligner [9] configured to allow up two mismatches, discard sequences with “N”s and return all optimal alignments. To perform the differential expression analysis between libraries, a normalization and statistics pipeline were applied using DEG-seq software [10] (confidence rate: 99%; cut-off: 0.01). RNA-Seq libraries and Gbrowse are available at http://bioinfo03.ibi.unicamp.br/eucalyptus/.

## Results and discussion

**Figure 1:**
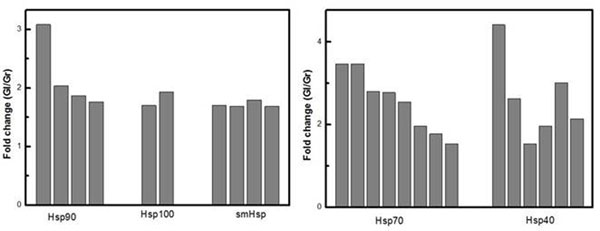
Molecular chaperones up-regulated in *E. globulus* xylem in comparison to *E.grandis* xylem.

In this study, we identified chaperones differentially expressed between *Eucalyptus* species. RNA-seq analysis revealed chaperones as smHsp, Hsp40, Hsp70, Hsp90 and Hsp100 between 1 and 3 fold up-regulated in xylem of *E. globulus* (Figure 1)*.*

**Figure 2:**
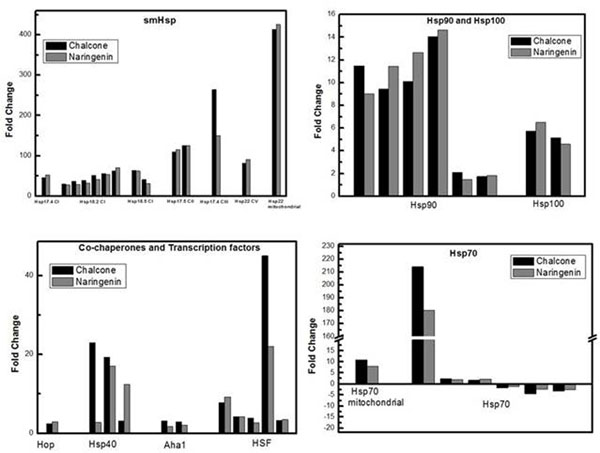
Molecular chaperones, co-chaperones and Heat shock transcription factors are up-regulated in *E. urograndis* narigenin-chalcone (black bars) and naringenin-supplemented (gray bars) in comparison to control group.

*Eucalyptus urograndis* narigenin-chalcone or naringenin supplemented also presented molecular chaperones highly expressed (Figure 2). Recent findings reported that supplementation with these flavonoids can inhibit lignin biosynthesis, and change lignin content and composition [10].

SmHsp family is active in a wide range of environmental stresses, including heat, cold, drought, salinity and oxidative stress [11]. Gion et al [4] noted smHsp accumulation in wood forming tissues, and suggested a role in extending the cell wall thickening phase during xylogenesis.

Hsp70 family acts in protein refolding, translocation, and facilitating the degradation of unstable proteins, directing them to lysosomes or proteasomes [12].

Hsp90 family has a select group of substrate proteins as polymerases and kinases [13]. In*A. thaliana* some kinases are required for optimal cell elongation, which is important for plant growth and vascular system formation [14].

We have successfully identified chaperones with higher expression on *E. globulus* xylem and in *E. urograndis* flavonoids-supplemented plants, which provide evidences to link xylogenesis and chaperone expression. Those findings are crucial on helping to elucidate the role of chaperones on plant development, stress response and wood formation.
